# Supplementary material for: “I don´t put people into boxes, but…” A free-listing exercise exploring social categorisation of asylum seekers by professionals in two German reception centres
Source: PLOS Glob Public Health. 2024 Feb 23;4(2):e0002910. doi: 10.1371/journal.pgph.0002910 (PMC10889701; doi:10.1371/journal.pgph.0002910)
Supplement: S1 File — (PDF) [file pgph.0002910.s001.pdf]

S1 File. Codes of the free-list analysis and assigned super-categories

Codes that are not self-explanatory or that summarize certain statements are provided with quotations. Statements that were assigned to a code, but slightly differed in wording from it are intentionally provided as quotes.

**0 = nation conglomerate or region**	
List-Item/Code (pseudonymized)	Comment/Quotes
nations1	
nations2	
nations22	specific region of geographical conglomerate nations2
nations3	
nations4	
nations5	
nations6	
plusnations	If the guiding distinction is not the region but another item (e.g., "there are economic migrants for example from nation3") the number is attached to this descriptor: economic migrants + plusnations3

**1 = nationalities**	
List-Item/Code (pseudonymized)	Comment/Quotes
nation1	
nation2	
nation3	
nation4	
nation5	
nation6	
nation7	
nation8	
nation10	
nation11	
nation12	
nation13	
nation14	
nation15	
nation17	
nation18	
nation19	
nation20	
nation22	
nation23	
nation24	
nation25	
nation26	
nation27	

nation28	
nation29	
nation30	
nation31	
nation32	
plusnation	Nation named, with another characteristic as primary distinction (same as described above)

**2 = ethnically defined collectives**	
List-Item/Code (pseudonymized)	Comment/Quotes
ethnicgroup1	
ethnicgroup2	

**3 = religions**	
List-Item/Code (pseudonymized)	Comment/Quotes
religion	Religion in general – seen as factor ("There are people with different religions…")
religion1	
religion2	
religion3	
religion4	
religion5	
plusreligion	Specific religion named, with another characteristic as primary distinction (see box 0 and 1 above). Example: "There are people that are very nice to their family, I encountered those for example when they follow religion2" = nice to family + plusreligion2

**4 = socio-demographic variables and attributes**	
List-Item/Code	Comment/Quotes
nationality	Nationality if it is mentioned as a factor in distinctions, without naming specific nationalities, e.g. "I distinguish people by their nationality"
male	
youngmale	Combined category, since it emerged in the data in the majority of cases in this combination
female	
young	
old	
children	
educated	
uneducated	
urban	

rural	
married	
single	
singlefemale	Combined category, if emerging in the data together
singlemale	Combined category, if emerging in the data together
alone	
alonefemale	Combined category, if emerging in the data together
family	
pregnant	
violentlypregnant	Combined category, pointing to pregnancies resulting from rape or other forms of sexual violence. Anchor quotes: “Nations4, I ask myself: How can a religious nations4 woman who is alone here be pregnant, the trafficker?”(S3D); “Single woman from nations2, […] why do they go alone? They sometimes get children from traffickers (raped or bought passage this way) […].” (A1D)
new mothers	
many children	Families or single persons having many children
disabled	If emerging without further reference to medical condition or care, seen as socio-demographic variable

**5 = flight motives and their classification**	
List-Item/Code	Comment/Quotes
real refugees needing help	“Real refugees who need our help” (F1D), “people seeking help, who really need it (L3D)
religious refugees	Flee because of their religious affiliation, fear of persecution
economic refugees	“The percentage of economic refugees is high.” (S2D); “Different reasons for fleeing (nation2s - mostly labour refugees, fleeing from living conditions - without being politically persecuted […]”(A1D); “Economic refugees - poverty, sometimes severe poverty” (D2D)
searching for a better life	Looking for better opportunities (without any negative connotation in the data): “want better chances in life (work and live)” (R2D); “Those-who-want-better-lives (most have similar desires to us, family/children, little house, job, car” (R2E)
war refugees	“Nation14, war, dictator!”(S4E); “[…] there is war in their country, then they come here” (S6D)
political refugees	“If there are political reasons, they say next to nothing anyway. They cover up everything […]” (D1E); “People who escaped in danger of their lives (e.g., because they printed leaflets)” (R2E)
health seeking migrants	Looking for existing or better medical care, hoping to be healed in another country: “Those who come for medical treatment, because it is too expensive in their

	country” (L2D). In 9 of 15 cases this is reported with a negative connotation, often using the term “medical tourism”. Example quotes: “nation22s, they come because of medical tourism, then they leave again” (L1D); “they get told […] if a surgery costs €10,000, if you [don't] have money, go to Europe.” (D1D)
fear of violence	“Revenge in family; fled, woman is afraid in nation4/nation17” (D3D); “[…] who have experienced violence (are in danger of being raped or have been, or of being killed)” (A1E)

**6 = motivations for Germany**	
List-Item/Code	Comment/Quotes
system exploiters	“[…] looking for benefits/advantages” (A1D), “want to exploit the German system” (D1D), “social tourism” (R1D)
want to stay	
want to leave	Referring to asylum seekers who want to go back home or move on to another country

**7 = imagined prospect of staying in Germany**	
List-Item/Code	Comment/Quotes
good perspective to stay	In most cases, the code was listed. Anchor quote which was not exactly the code: “those with a prospect of protected status” (R1D)
poor perspective to stay	“Lack of perspective due to the lack of prospects of staying in the country” (R1D); “young economic refugees, those will be the first ones to be deported” (S4E); “from nation2, nation 5 and nations2 they have no prospect of staying” (S2D); “some want to integrate, but will be deported anyways” (A1D)
save countries of origin	Additional quote that is not exactly the code: “nation5 is a safe country” (D2D)

**8 = migration patterns**	
List-Item/Code	Comment/Quotes
multiple migrations	“People who have been to other places before [like Italy] can't be sent back, come right back” (S3D); “re-entered the country” (A1D); “euro bouncer” (A2D)

9 = bad experiences and victimhood	
List-Item/Code	Comment/Quotes
victims of violence	
victims of sexual violence	“[…] have been raped” (A1D); “[…] sexually abused” (D2D)
victims of torture	
victims of human trafficking	Additional – difficult to place – statement, that has been put in this category: “How can it be that a young woman, who has just arrived is than picked up by a car nearby by a nation15 and brought back…?” (S3D);
prostitution	
victims of FGM	
victims of discrimination	“Ethnicgroup1 come because are not seen as human beings, get no work, no school - right to nothing” (D3D)

10 = references to culture	
List-Item/Code	Comment/Quotes
oppression of woman	“The man has the say, he talks“ (R1D); “woman is like an animal, for some religion1s” (S3D); In some countries, women are worth nothing, which sometimes takes some getting used to” (F1D)
no oppression of woman	Quote: “nations2 – have respect for woman”
neglecting parents	“[Those who take care of children], those who let them run free” (S3D); “Children are sometimes treated carelessly, e.g., they simply walk alone, or mother walks on, child far behind” (F1D)
caring parents	
behaving differently than in their country	“Drink alcohol once a month, which is forbidden in their country” (S6D)
closer to my culture	“[Degree of foreignness] Different cultural backgrounds: From nation7 (academic), with whom I can talk immediately (culturally and educationally similar) (A2D) [quote continues below]
further from my culture	…to nation1, who comes here without underwear in her robe, archaic, where for me is something completely foreign” (A2D); “nations2, where I culturally feel insecure, because I know nothing about it.” (A1E)

11 = with relation the locality	
List-Item/Code	Comment/Quotes
shorter there	“New” (T1E, L1E), “quickly gone” (S3E), “only a few months there” (S4E)

longer there	"Those who have been here longer (little bit of a personal bond)" (T1E); "have been there as long as me, you already know them" (T5E)
often met and/or familiar	"People who come again and again" (S1D); "every day the same people" (S4D), "long-term care" (R3D)
not often met and/or unfamiliar	Different wording, assigned here: "only one time contact" (R3D)
waiting	"you have nothing to do, do not work, have time, just have to wait" (D3D)
understand system and rights	"Those who wait in line and others who flash their badges (me now!) where there is an understanding of the system and where there is no understanding of the system – a lack of understanding in the daily live in the office" (T3E) [second part assigned to next code]
do not understand system and rights	"Those who appeal, those who can, and those who don't, perhaps because they can't assess the possibilities and their rights." (T3E)
residents	Inhabitants of the reception centre
guests	People who are visiting others in the centre
car	People who own or use a car (meaning enter the facility with it)
no car	Entering the facility without a car
conflicts	Additionally assigned: "Those who can not share rooms" (H1E);
different disciplines	"They come visiting different (medical) disciplines" (S4D)

**12 = language**	
List-Item/Code	Comment/Quotes
with translator	Access to translation and its effects described "first thought: do I need translation?" (A2D) "translated conversations" (A1D); "they come with translators, kids, neighbours" (T3E); "which translator is involved changes the entire conversation" (A2D); "I feel safe when a good translator is present and insecure, when it is complicated." (A1E)
without translator	"Is there no translator, and I have to improvise?" (A2D); "some say yes, even if they do not understand" (T3E); "I am glad, when somebody can speak German well, or English" (A1E)

**13 = manners and behavior**	
List-Item/Code	Comment/Quotes
polite and friendly	"Say please and thank you" (F1D); "smile, are friendly" (L1D); "polite and appreciative" (R1D); "nice ones, you

	like to see" (T1E); also summarises references to people being sweet, respectful or helpful
impolite	Pointing to rudeness or not respectful behaviour "do not greet, do not say please or thank you" (T1E); "have no respect" (S3E); "are snotty and grumpy" (F1D) "Behaviour sometimes extreme when in company, become calm and nice without their peers" (T2E);
gift-giving	"Those, who always/often bring a present" (T1E, T3E)
calm	"[…]: calm, not aggressive, ask, pray" (S5D); "[...] no quarrel, no one has ever had a problem with them" (S5D)
loud	
tense	"Jaw muscles tense, eyebrows, I perceive that unconsciously" (D1D)
aggressive	The exact term has been used by most participants, even if definitions differed: "[…] are not aggressive, like Nations4, that want to achieve everything trough violence" (S5D) "Aggressive body posture" (L1D); "Aggressive behaviour in some cases (want to get something for free)" (S2D); "Aggressivity, very emotional, [they carry what is going on where they come from]" (S5D);
honest	
deceiving	"Liars. False identity assumed, for example, but understandable, because they would not have gotten out of certain countries otherwise" (R2D); "some who lie a lot" (S1E); "Those, who free ride, with the stream of refugees, claiming they come from nation19 [but its not true] (A2D); "People who cheat: Don't want a transfer, say I'm mentally ill" (S2D)
flirting	"[…] try to flirt" (T3E); "pick-up lines" (T3E); "[…] who quickly misunderstand [my signals as romantic interest]" (T3E)
nice to family	"Loving interactions with relatives" (L1D)
not nice to family	"Dismissive interactions with relatives" (L1D)
clean	"Very clean ones (one family here is extremely tidy/clean) (G1E)
unclean	"Leave [rubbish] and do not want to clean up after themselves" (F1D); "Cleanliness/hygiene, people are not taught, you don't learn it in school." (SE3D)

**14 = attitudes**	
List-Item/Code	Comment/Quotes
insecure	Also: "do not dare to ask for help" (T2E); "those who are not confident" (T3E)

confident	Self-confident, “extremely self-confident” (T3E); (“clear” [say clearly what they want] (D2D);
resigned and lethargic	Resigned to depressed [because of many obstacles] (D3D); “Lethargic ones (must not work, "allowed nothing, nothing, nothing" (G1E) “Resigned ones, with no expectations that they can be helped” (L5E)
indifferent	[…] some, who don´t give a damn” (T3E);
warm and open	“Hearty [people]”[1] (L2D); “Those who come to talk” (T3E); “very open, tell something” (T3E); “quickly in personal contact” (T3E)
distanced and withdrawn	“Unsociable [people]” (T3E); “those who don´t tell much” (D1E)
demanding and expectant	“Discerning [people]” (e.g., L3D, R1D, R3D, T1E, T4E, G1E); “a lot of them want something” (F1D); “have a demanding nature” (A1D); “want to get everything out of it, want to put you under pressure” (A2D); “those who still want more” (T3E) “demanding, this belongs to us – we take it” (T4E); “extremely high expectations” (L5E);
scrambling and impatient	“Rushing me, can't you go faster, everything is urgent” (T3E); “impatient and quick-tempered” (L3D); “jostling ones, want to get in first” (S4E)
thankful	
not thankful	Additional quote assigned here: "I saw those things I brought [as a gift/donation], were broken"(F1D)
adapted and subordinate	“Insightful [people] [realise, that things are how they are] (L3D); Those who queue up nicely and wait” (T3E); “come during opening hours” (T3E); “fastening their seatbelts voluntarily” (F1E)
not adapted and insubordinate	“Unwilling to accept, struggling/fighting” (T3E); “don't stick to the rules [...], grumble, claim I have no say” (F1D); “those who do not take no for an answer (S2E); “unadjusted and unruly” (F2D)
audacious	“Rascals/smarty pants” (S2E); “insolent and impudent” (G1E); “High-flyers, scoundrels and bon vivants” (D1E); “rather cheeky, if you explain the rules, they want to leave right away” (S3D)
jealous	Jealous and/or envious people
imputing racism	“Those who think, others are racists” (S5D); “saying I am a racist, a nazi” (F1D)
moody	
unhappy	“Happy-to-be-here, is no one” (H1E)
coping	“Coping greater for some, although also traumatised” (L1E)
lesser coping	“Severely traumatised, cannot take the help” [in the sense, that they cope less good, so that it does not work] (L1D)

[1] In German possible to state a capitalized noun/substantive to describe a type of person

dependent	“Those who become totally dependent” (L1D); “Help-less [people], can't cope, need another kind of care” (L5E)
independent	“Independent patients […]” (L1E)
hopeful	“Hopeful [people]” (L5E)

**15 = attribution of own reactions**	
List-Item/Code	Comment/Quotes
annoying	“Annoying people, you don’t like to see” (T1E); “un-seemly [annoying] way of pick-up” (T3E); “Those who annoy you - where everything goes round in circles – [you have to explain rejections repeatedly], they don’t want to believe it” (L5E)
exhausting	“Exhausting [people]” (L5E); “Difficult patients (I can't go on, I try to give and talk, I can't [do it any longer]” (L1E)
fear inducing	“Those I don't want to have here, those I don't want to meet here” [statement made in the context of anxi-ety] (A2D)
gender awareness	“Women's issues (pregnant – unclear, human traffick-ing/circumcision – possibly only female counselling desired)” (R3D); “You're always a bit nicer to women – as a woman –- because you want to assert yourself with men: In some cultures I have no say” (T1E); “Some do not want to be treated by women, do not dare to ask for help from them” (T2E); “You should pay attention to how you are dressed as a woman (no open décolleté)” (T4E)
burdening	“Patients I take home (their fate, their story, where I don't know what happens next [I had to tell a woman she was HIV positive, after she had been raped [on the flight]” (A2D);
triggering compassion	“Those I feel sorry for, I want to do something good for them.” (A2D); “Nice ones, that I feel very sorry for” (A2D)
not trustworthy/reliable	“When I meet them in town – not in the clinic – I think differently about them [have more reservations].” (A2D); “Some are quiet/nice (because they want something from me, but I don't trust them)” (D1D)
trustworthy/reliable	“Reliable [ones], can be relied on” (T3E);
fuss-free	“uncomplicated” (T3E); “unpretentious/modest” (L5E)
injustice	Something unjust happens to people in the eyes of the descriptor: “[...] others integrate job/apprentice-ship/training -> then they are deported, that is un-just/unfair” (F1D)

**16 = appearence**	
List-Item/Code	Comment/Quotes
clothes	“Clothes - chic/lumpy, I know, mega superficial, but you still pay attention to it” (L1D)
posture	“Body posture […]” (L1D)
other physical characteristics	“Fingernails, I don´t know why but I look at everyone’s fingernails” (L1D); “differences in faces (bones under the eye, jaw muscles: tense/relaxed, eyebrow move-ment) […] I perceive this unconsciously and know who I'm dealing with/what people are like” (D1D)
colour of skin	“Blacks/whites” (L2D); “Blacks, I can mostly recognise, assign [a nationality to]), whites are harder to assign” (S4E)

**17 = health and illness**	
List-Item/Code	Comment/Quotes
healthy	“Healthy people” (T2E)
(properly) sick	“Sick migrants” (D1E); “People who are sick, there are a lot, because here is [the local hospital]” (L2D); “Properly sick people” (L3D); “The kind, that really have something” (D3D)
faking illness	“80% fakers - want certificate (overslept [for their asy-lum interview] or transfer)” (L1D); "People who are [...] kind of phoney, I can tell when someone has something or has nothing” (D3D)
hypochondriac	“Those who think they are sick but actually have noth-ing” (L2D)
whole-body pain	“Many who state as afflictions: My whole body hurts” (L2D)
physically ill	
psychological issues	
traumatized	“Psychotraumatised people, need trauma therapy” (A3D); “Psychosomatic, post-traumatic – this group is much larger here than in private practice” (A2D)
mild diseases and needing general care	“[…] some only need pills” (S4D); “[…] have little things, sports injuries” (A5D); “normal, primary care needed” (L1E); “Fit [people], cough, cold, fever, only come when they are actually sick and tend to take care of themselves” (L2E)
acute disease and pain	Usually the term “acute” was used, one other exam-ple: “pain patients” (A5D) was also assigned here
chronic disease	All mentioned chronic diseases were assigned here (kidney, metabolism, asthma) as well general mention of chronic disease: “Those with chronic illnesses that can lead to further deterioration” (A3D); “Chronically ill, with nothing I can do” (A3D)

oncology	“Onco-patients” (L5E)
seriously ill	“Some are very sick” (S4D); “Seriously ill patients: complex leukaemia, often multimorbid, breast cancer with chemo/radiation, sometimes also terminal care. Many are completely cared for here, in cooperation with the clinic, etc.” (L1E)
drug consumers and addicts	“[…] often addicted to drugs” (L3D); “having drug problems” (T2E)
Tuberculosis	
HIV/Aids	

**18 = health care related knowledge and behaviour**	
List-Item/Code	Comment/Quotes
not snivelling	“Not at all snivelling […]” (A1D); “[…] are tough” (L1E)
snivelling	“[...] more snivelling […]” (T2E)
poor health literacy	“Understanding the treatment is often difficult, in [cardinal direction] countries, they give antibiotics on mass for every cold, here we have to explain” (A4D)
higher health literacy	“Patients with different levels of prior knowledge, urban […], education level” (L2E)
wanting certificates	“Those, who need certificates” (A2D); “Patients who are only pursuing their goal, as far as staying here is concerned, looking for a reason to be allowed to stay here.” (L3D)

**19 = criminal and conspicuous behavior**	
List-Item/Code	Comment/Quotes
criminals	
drug dealers	“You can go to a room and give someone 10€ because it is known that he is dealing” (S3D)
black market actors	
drug consumers and addicts	“[…] often addicted to drugs (L3D); “drug addicts – young men, that’s also a group […]” (A4D)
domestic abusers	

**20 = integration**	
List-Item/Code	Comment/Quotes
integration and/or working effort	“[…] want to live here, go to school, work (S3D); “refugees who work” (S6D); “willing to work” (A2D); “[…] integrated ones, start to learn German/English themselves” (L1D); “motivated ones, learn the language, look for a job” (L3E); “integrate quickly, want to have their training recognized […] (D1D)

no integration and/or working effort	“%80% really seek our help, 40% of them are deductible, because they do not want to integrate” (F1D); “some have been here long and still speak German poorly” (T3E); “some are grateful, but without the wish to integrate” (T1E); “[…] they work the least” (S3E)
good integration chances	“Those who can integrate easily, not only language wise” (A2D)
bad integration chances	“[…] many of them are unskilled workers” (A1D); “[…] lack of perspectives […], are aware of their poor prospect of staying, some do not speak German even after a long time” (R1D)

**21 = counselling topic (social work)**	
List-Item/Code	Comment/Quotes
seeking asylum advice	
questions about transfer	

**22 = structural issues and other topics**	
List-Item/Code	Comment/Quotes
structurally no treatment possible	“[handicaps, chronic illnesses] […] very depressing. I can deliver bad news, "wait for your status" (before that I can't do anything for them, no expensive examinations (I have to ask, need permission from [the government authority], only if they agree... […] for chronic diseases only 'if it makes sense'” (A1D); “chronically ill, where I am not able to do something” (A3D); “Germany can not help everyone, has own problems […], I tell them, we can not do so much, it´s better they know” (D1D); “lack of structures for mentally ill ones [bipolar disorder, …], I worry, how she should be cared for here.” (A4D)
vulnerable and in-need-of-protection	“There are highly vulnerably [ones], in need of protection” (A3D); “those who need help” [in general] (L2D)

**23 = refusal to generalize and discriminate**	
List-Item/Code	Comment/Quotes
refusal to generalize	"I consciously try not to pigeonhole people" (R2E): "I have tried not to let the pigeonholing get to me... if I only think in terms of divisions - I am in the wrong place... then I am so prejudiced... that I can't do it any more" (T5E); "The question is difficult, reinforces prejudices [...] I don't classify, you have to make up your own mind" (A3D); "In the forest [everything is] green,

	one tree [is] sick: there are many good, few bad." (S6D);
equal treatment	"I am nice with everyone" (D1D); "You have to treat everyone the same, adjust to the situation depending on how they are/behave [...] (S2E); "I have love for all, am not a racist" (S5D); "I do not ask why they are here, I am here […] to help everyone in their lives" (A3D)

**24 = referral to third party perceptions**	
List-Item/Code	Comment/Quotes
public and media perceptions mistaken	"Reality is different from the media" (S5D); "[…] from the media you think they have negative attitude/are hostile, but they are polite and courteous" (T3E); "My personal opinion about mass media in Germany... people don't get enough information (nice clothes, taxi, mobile phone) is what they see with asylum seek-ers" (D1D)
