# Supplementary material for: “I don´t put people into boxes, but…” A free-listing exercise exploring social categorisation of asylum seekers by professionals in two German reception centres
Source: PLOS Glob Public Health. 2024 Feb 23;4(2):e0002910. doi: 10.1371/journal.pgph.0002910 (PMC10889701; doi:10.1371/journal.pgph.0002910)

S1 Comparison. Flame Analysis 3 (including national/regional specifications) ordered by frequencies

Table 1C. Health professionals (n=14: physicians, nurses, medical assistants)

Original Name	Occurrence Number	Frequency	Summed Ranks	Average Rank	Sutrop Index
health seeking migrants	8	57.14%	94	11.750	0.049
traumatised	6	42.86%	63	10.500	0.041
demanding and expectant	6	42.86%	65	10.833	0.040
wanting certificates	6	42.86%	81	13.500	0.032
psychological issues	6	42.86%	100	16.667	0.026
educated	5	35.71%	73	14.600	0.024
searching for a better life	4	28.57%	17	4.250	0.067
youngmale	4	28.57%	31	7.750	0.037
integration and/or working effort	4	28.57%	32	8.000	0.036
plusnation19	4	28.57%	42	10.500	0.027

Table 2C. Security (n=10)

Original Name	Occurrence Number	Frequency	Summed Ranks	Average Rank	Sutrop Index
not adapted and insubordinate	4	40.00%	12	3.000	0.133
economic refugees	4	40.00%	14	3.500	0.114
adapted and subordinate	4	40.00%	16	4.000	0.100
calm	4	40.00%	23	5.750	0.070
aggressive	4	40.00%	28	7.000	0.057
plusnation5	4	40.00%	70	17.500	0.023
equal treatment	3	30.00%	20	6.667	0.045
integration and/or working effort	3	30.00%	22	7.333	0.041
criminals	3	30.00%	27	9.000	0.033
demanding and expectant	3	30.00%	32	10.667	0.028

Table 3C. Other staff (n=26: social work, translation, administration, facility management)

Original Name	Occurrence Number	Frequency	Summed Ranks	Average Rank	Sutrop Index
polite and friendly	10	62.50%	68	6.800	0.092
demanding and expectant	8	50.00%	51	6.375	0.078
female	8	50.00%	69	8.625	0.058
integration and/or working effort	8	50.00%	87	10.875	0.046
aggressive	6	37.50%	63	10.500	0.036
thankful	6	37.50%	64	10.667	0.035
political refugees	5	31.25%	32	6.400	0.049
male	5	31.25%	49	9.800	0.032
oppression of woman	5	31.25%	56	11.200	0.028
economic refugees	4	25.00%	25	6.250	0.040

Figure 1C. Comparison of super categories (health professionals, security, other staff)

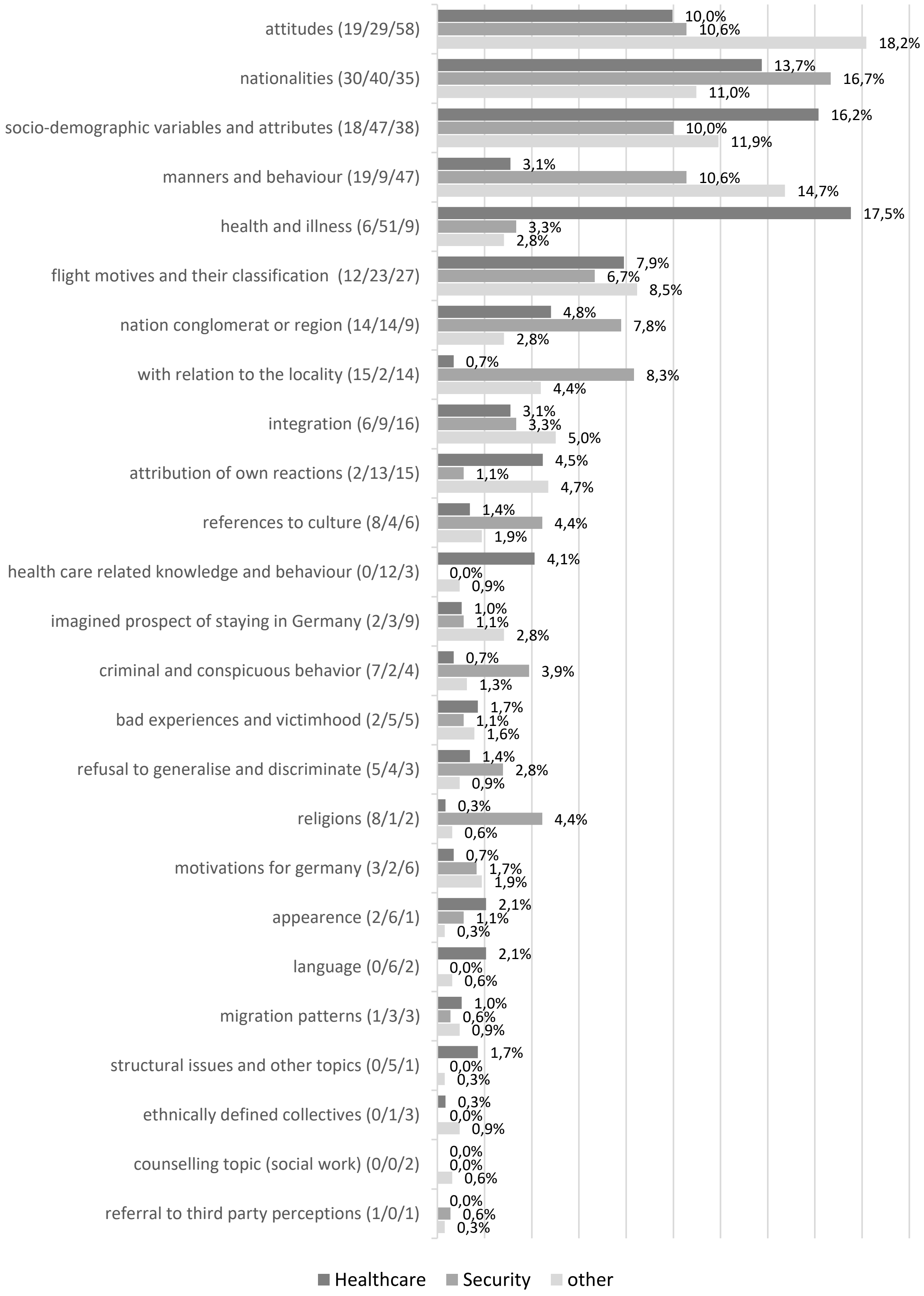

Figure 2C. Comparison of focus categories (health professionals, security, other staff)

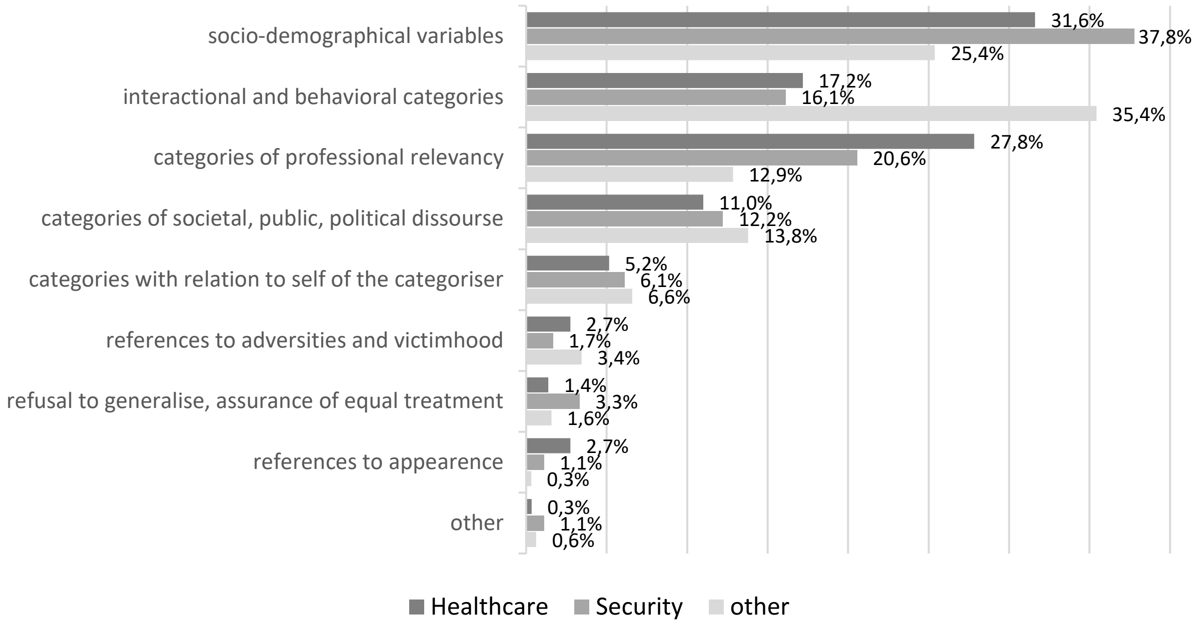

Supplement: S2 File — (PDF) [file pgph.0002910.s002.pdf]
