# Supplementary material for: “I don´t put people into boxes, but…” A free-listing exercise exploring social categorisation of asylum seekers by professionals in two German reception centres
Source: PLOS Glob Public Health. 2024 Feb 23;4(2):e0002910. doi: 10.1371/journal.pgph.0002910 (PMC10889701; doi:10.1371/journal.pgph.0002910)
Supplement: S1 Table — (PDF) [file pgph.0002910.s003.pdf]

**S1 Table. Descriptives of two rounds of the free-list analysis in Flame**

| | Exclusive nation specifications | Inclusive nation specifications |
|---|---|---|
| Number of lists | 40 | 40 |
| Average length of lists | 16 | 18 |
| Minimum length of lists | 4 | 4 |
| Maximum length of lists | 31 | 38 |
| Number of unique social categories | 165 | 219 |
| Total number of items analysed in Flame | 624 | 701 |
| Total number of items including duplicates | 790 | 790 |
