# Supplementary material for: “I don´t put people into boxes, but…” A free-listing exercise exploring social categorisation of asylum seekers by professionals in two German reception centres
Source: PLOS Glob Public Health. 2024 Feb 23;4(2):e0002910. doi: 10.1371/journal.pgph.0002910 (PMC10889701; doi:10.1371/journal.pgph.0002910)
Supplement: S2 Table — Incl. duplicates, with/without nation/region specification. (PDF) [file pgph.0002910.s004.pdf]

S2 Tables. Top 10 absolute frequencies. Inclusive duplicates, with and without nation/region specification

1. Nation specifications included

Freq.	item
22	demanding-and-expectant
21	polite-and-friendly
16	aggressive
16	integration-and/or-working-effort
15	economic-refugees
15	health-seeking-migrants
13	female
13	not-adapted-and-insubordinate
12	calm
11	thankful
10	adapted-and-subordinate
10	educated
10	no-integration-and/or-working-effort

2. Nation specification excluded

Freq.	item
64	plusnation
37	nation
22	demanding-and-expectant
22	plusnations
21	polite-and-friendly
20	nations
16	aggressive
16	integration-and/or-working-effort
15	economic-refugees
15	health-seeking-migrants
13	female
13	not-adapted-and-insubordinate
12	calm
11	thankful
10	adapted-and-subordinate
10	educated
10	no-integration-and/or-working-effort
