# Supplementary material for: “I don´t put people into boxes, but…” A free-listing exercise exploring social categorisation of asylum seekers by professionals in two German reception centres
Source: PLOS Glob Public Health. 2024 Feb 23;4(2):e0002910. doi: 10.1371/journal.pgph.0002910 (PMC10889701; doi:10.1371/journal.pgph.0002910)
Supplement: Attachment — Submitted filename: AdressingReviewer1and2pointsFINAL.docx [file pgph.0002910.s006.docx]

LETTER TO THE REVIEWERS

Dear Reviewers,

thank you for your interest and time as well as for your valuable and constructive feedback on our manuscript. Your comments and suggestions helped us to improve the article and make it more accessible to readers from different scientific fields. We have addressed all your comments and will refer to each of them below.

Reviewer #1: Dear authors,
thank you very much for this intriguing contribution to the growing body of literature on social categorisations of (forced) migrants. While most contributions to this research field in the social sciences are of a conceptual or theoretical nature, the present article offers an attempt to empirically grasp the way how asylum seekers/refugees are categorised, how those social categorisation processes can be understood in light of ongoing policy and public debates, and what consequences those categorisations might have with regards to the perpetuation of public discourses about forced migration.
Given the very scarce empirical research into categorisation processes of (forced) migrants, the explorative approach of free-listing is an appropriate way of generating knowledge about the way how forced migrants are perceived by different groups of professionals. The mixed-methods design is described in an appropriate way that also shows that the authors are aware of the limitations to this approach.

Thank you very much for this feedback.

The text is well-written in plain English. The conclusions drawn from the results are generally plausible, although some further explanations might be needed, as I will account for in more detail below.
In general, I recommend publication of this article, which offers insights that might lead to a better understanding of the relevance of social categorisation processes in the context of forced migration. However, I would like to offer some suggestions to the authors for consideration:
1) The results show very distinct ways of categorising forced migrants. Some are related to their perceived behaviour (thankful, polite, aggressive, etc.), some relate to their status ("economic" refugees, legitimate refugees, bogus asylum seekers, etc.), and others relate to their national or ethnic background. Whereas the last two categories clearly refer to forced migrants, one might wonder if the first category refers to patients in healthcare or people in general. Maybe if asked "what kind of PEOPLE are there" the same answers from the first category could have emerged. Obviously, it is impossible to answer this question in retrospect, however it might be worth a reflection in the paper. These freelistings might imply that the professionals think about asylum seekers / refugees in those categories, it might also be that - particularly medical practitioners - in general think about patients in these categories regardless of their migration or non-migration status. The latter would also be an interesting finding but leads to different conclusions (maybe the respondents who listed those categories do not really distinguish between asylum seekers / refugees and non-mobile populations).

Indeed, you raise a valid point. The significance of categories that evaluate gratefulness or fretfulness or in general “behaviour and attitudes” surely also extend to categorisations of German patients. We have now provided references from other – mainly qualitative studies – on such categorisations and reflect on the significance of such categorisations in refugee care settings in more detail within the paper (see lines 691-713).

We believe that the application of such categories to refugees in reception settings may have or can take on a different significance. Based on field experience and literature, there seems to be a specific image of the "suffering refugee" that is expected to be quiet, dependent, and thankful. In order to fully benefit from the dedication of professionals, refugees seem to be expected to conform to this image. Unfortunately, a high level of dedication of professionals is frequently required for them to receive adequate care, as – for example - their healthcare access is restricted by law, which means additional effort for professionals regarding the bureaucracy of care financing. Those who provide care rarely receive (social) recognition for their work from their employers or the government responsible for funding basic services. On the contrary, caregivers often face structural and bureaucratic obstacles in their efforts to uphold their professional values. There is a feeling that one is providing care "despite everything," against all odds, sometimes combined with the feeling that they would not have to or even shouldn't – so they help out of the goodness of their hearts, because they see the need and suffering. In return, they expect (and receive) “nothing more than a little gratitude.” If even that is not granted (by the patient) they might feel betrayed in their efforts to help (see also “reciprocity” as deservingness dimension).

(Even or especially) a self-perception as steadfast representative of the own profession, fighter for international justice and equal health opportunities, or even benevolent saviour can be called into question when a patient does not fit the expected victim mentality and does not enact vulnerability but instead demands certain things vehemently or even aggressively, such as an MRI for back pain, HIV medication, or chemotherapy – these intentionally different examples can elicit a similar social response of rejection or repulsion from the professional, almost irrespective of the severity of the illness. Interestingly enough, in this context, the opposing category to the grateful patient is not the ungrateful one but rather the demanding patient. In our opinion, within the refugee context, these social imaginaries– which also guide social behaviour in regular healthcare settings – can take on a different intensity and have more significant consequences regarding refugee patients, due to the structural disadvantages of the patient collective, the power imbalance and respective higher degree of dependency of patients on the good-will of these specific (random) professionals on site to receive proper care. Patients and clients can not as easily as other patient ask someone else, outside the facilities. We could hypothesise, that might even contribute to the perception of being demanding, since, they (have to) ask, these same professionals (maybe more than ones) if their needs were not met. Fact is, they might not as easily access another facility outside the reception setting, to get what they need and/or want, like majority patients would (e.g., due to a lack of knowledge about German healthcare laws and system and the structures of care provision on site). Therefore, social mechanisms and evaluations, like the described ones may have a higher impact on access to healthcare services, that is the case within the regular system.

2) Given the distinct groups of professionals that were included in the free listing exercise and the potentially different outcomes, I am not sure if I agree with the authors that a distinction of the results by groups should be avoided. In order to have higher numbers of respondents in each group, groups could be merged, for instance into health personnel, support personnel and security personnel.

We re-calculated our numbers for the different groups you propose. The additional results you find now in the Supplementary (S4 Comparisons). The Flame Analysis of frequencies of mention provides some interesting results on distinctions in social categorisations of those groups, which we now mention in a new chapter of the results section. We also tried out to order by Salience, but this did not provide meaningful results for health professionals and security, since in this small sample sizes items that were just mentioned once, where high in the lists, because they were mentioned first. We also conducted the secondary, qualitative coding (super and focus categories), which includes duplicates. We advise caution in interpreting the results since multiple mentions of the same category by singular participants has a heightened impact on this analysis. For example, the impression, that medical personnel seems less influenced by behaviour than by professional considerations should be considered together with the Flame analysis results (with: health seeking migration, psychological issues *and* demanding behaviour most frequently mentioned by health professionals). Larger comparative studies would be interesting and advisable to look deeper into distinct categorisations of different professional groups.

We also changed the title of our manuscript, so the aim to explore social categorisations of the members of different professions that take care of asylum seekers in reception is made more transparent.

3) Although the concept "deservingness" seems appropriate in this context, it needs a bit more of an explanation about the way how it emerged in this context (from the data, from the literature, from public discourses), hence why is it used to embed the findings in the discussion part but not introduced in more detail, for instance in a theoretical background section or in a literature review section? It might help the reader to appreciate the reference to this concept more if its meaning for understanding the results were introduced in a bit more comprehensive way.

As you proposed, we now introduce the concept of deservingness in a new section within the introduction.

We encountered the connection between social categories and deservingness negotiations during the field phase. Especially for government administrative staff, the legitimacy of reasons for fleeing was closely linked to their prioritisation decisions regarding granting or denying requested healthcare services. The healthcare personnel perceived heightened restrictions when dealing with individuals that had been classified as economic or medical refugees, and/or came from certain countries that were considered as safe. Probably partly in response to that and partly due to own political opinions, they also engaged in moral discourses on deservingness, which were linked to various medical, logistical, political, and social questions: How severe is the illness? Is immediate treatment necessary or can it be postponed? Would my request for cost-coverage be approved for this patient? Why is someone here? What do they bring to my country? Is the person behaving appropriately? Since we are in the process of preparing another article (for BMC Health Equity Studies) where these prioritization discourses, processes and criteria will be discussed in detail, we did not introduce such partial findings from the broader ethnographic study in this article.

**Reviewer #2:** I have carefully reviewed your manuscript and would like to provide a constructive and respectful peer review. Overall, I appreciate the effort put into this research and the significance of the topic. Here are my comments on each segment of your paper:

Abstract: The abstract provides a concise overview of your study; however, it could benefit from a stronger connection with your manuscript. It is crucial that the abstract mirrors the research questions and findings presented in the manuscript to ensure coherence. Additionally, highlighting the positive results and their relevance to the research questions would make the abstract more impactful and of interest to readers.

Thank you for the feedback. We changed the abstract, aligning it better with the research questions and presenting more detailed positive results now.

Introduction: The introduction effectively sets the stage for your research by providing background information and linking it to the importance of social categorization in the context of asylum seekers and immigration. You successfully establish the relevance of social categorization with references to previous literature. To enhance your argument, **consider elaborating on the interaction between political and societal discourse and its impact on social categorization, as this seems central to your study**. Furthermore, **clarifying the connection between your two research questions and exploring the potential implications of discrimination and public discourse on social categorization** would strengthen your introduction. It would also **enhance the introduction to provide background information on deservingness**.

As you proposed, we now provide background information on the concept of deservingness in a new section within the introduction. We also added a second introductory chapter where we elaborate on the interaction of political and societal discourse and social categorisation. The question about the interaction between political/societal discourse and social categorisation of individual professionals is a good one and could be properly addressed in a sociological or socio-psychological book. A large, special research area funded by the German Research Foundation (DFG) is currently dedicated to these and related questions on human differentiation (<https://gepris.dfg.de/gepris/projekt/442261292?context=projekt&task=showDetail&id=442261292&>). The complexity of interconnected, interacting factors that mutually reinforce each other can not be entangled within the frame of this paper, but we now provide more reflections on this connection.

Materials and Methods: While I am not an expert in qualitative research, it is essential to ensure that your methods are clearly explained for the benefit of all readers. Consider **providing additional details about data processing and analysis, particularly in the context of multiple-step qualitative analysis and the three-step content analysis**.

Thank you. We now restructured the methods explanation to clarify the methodological approach in more detail and give more examples to better understand the qualitative analysis process (lines 270-346). We now also point more clearly to the Supplementary File S1 which provides a “code-book” that helps to understand a coding process better.

**Addressing the issue of generalizability** is important, and you might want to discuss the **limitations of applying findings from a single social unit to a larger population of similar units**.

We added the issue of generalisability to the limitations section (see lines 824-827).

It's commendable that you **mention the approval of your study by the university ethics committee.**

As we stated in lines 260-261 (chapter “study design”), the study was approved by the ethics committee of the Heidelberg University Hospital**.**

Results: To improve the clarity of your results section, **consider describing the variables presented in the tables to allow readers to interpret the tables independently**.

We describe our measures (Frequency 1+2, SIS, average rank, %tage) in the methods section. In the results section we only can describe the most important/most salient singular items. All items (also the ones that are not shown in the tables, since those categories were not used much) can be followed up on in the Supplementary SI File, in column 1. In column 2 we also provide some anchor-quotes and examples showing the continuum of statements that were condensed in an item, if they differed in wording.

Additionally, it would be beneficial to keep **the results section focused solely on presenting the results, without delving into the methodology used to obtain them, this should be mentioned in the methods section**. Avoid providing interpretations in this section…

Thank you for pointing that out. We deleted introductory, summarising references to methods from the results sections. We additionally made sure, there were no further interpretations provided with the quantitative results.

However, it is common to present some analytical remarks with qualitative findings (which we did in the last results-chapter). Especially in our case, where we only provide a (few) additional, qualitative insights, that could not be grasped fully within the quantitative calculations and results. Explanations have to be connected with the specific quotes, for the readers to understand the data better. Furthermore, we aimed to focus the discussion on two specific findings (demanding-and-expectant and economic refugees) to be able to discuss them in more depth.

…and present the results in the same order as your research questions were posed in the introduction.

The order of the research questions is:

1. What social categories are there?
2. What kind of social categorisations can be identified? (Super- and Focus-Categories)
3. How permeable are social categorisations to the public discourse on flight migration?

The order of results presentation is:

1. Social categories that were found
2. Remarks on the comparison of groups of professionals
3. Which social categories could be assigned to the public discourse on flight migration?
4. Kinds of social categorisations (super categories)
5. Kinds of social categorisations (focus categories).
6. Which proportion of the focus categories is referring to the public discourse?

We understand your feedback well and planned to change the order of the text accordingly, trying to put 2) and 5) in one chapter. However, we realized, that because of our multi-step analysis approach (first analysis: singular items/social categories *excluding* duplicates with Flame, second analysis: structuring, condensing analysis of broader themes, *including* duplicates with Excel) the changed order would hold the danger for findings to be misinterpreted by the readers, since the findings from 1) and 2) are based on another form of analysis and proportions should not be compared with the findings from 3-5.

Discussion: Start the discussion with a concise summary of your research's positive results and their alignment with the existing literature. To enhance clarity, use a consistent term to refer to social categorization throughout this section. Expanding on the relationship between **social categorization and public discourse**, in addition to the discussion of deservingness, would strengthen the overall argument. Frame the discussion in a way that ties your research findings to the existing literature.

We now use “social categorisation” instead of “differentiation”. We are now expanding on the relationship between social categorisation and public discourse in more detail in the manuscript. Furthermore, we now provide some additional literature that we tie to our findings.

Your acknowledgment of study limitations is appreciated, but consider presenting these **after discussing your findings** and **highlighting your study's strengths**.

Thank you, changed the order of the chapters accordingly.

Conclusion: In your conclusion, focus on summarizing your research questions and the corresponding findings. Ensure that your conclusions align with the scope of your study and avoid introducing new elements.

We now summarise our findings in the conclusion and point to the political instrumentalization and internal border work of professionals in reception setting, which we have introduced in the discussion.

In summary, your study presents valuable primary research, and the topic's relevance is clear. However, some improvements are needed in terms of clarity and organization in various sections of the paper. While I cannot confirm the novelty of your results, I encourage you to clarify and expand upon your methods, align your conclusions with your research questions, and address the points raised in this review. It is my belief that with these revisions, your manuscript has the potential for publication.
